# BPTF is required for c-MYC transcriptional activity and *in vivo* tumorigenesis

**DOI:** 10.1038/ncomms10153

**Published:** 2016-01-05

**Authors:** Laia Richart, Enrique Carrillo-de Santa Pau, Ana Río-Machín, Mónica P. de Andrés, Juan C. Cigudosa, Víctor J. Sánchez-Arévalo Lobo, Francisco X. Real

**Affiliations:** 1 Epithelial Carcinogenesis Group, Cancer Cell Biology Programme, Spanish National Cancer Research Center-CNIO, Madrid 28029, Spain; 2 Molecular Cytogenetics Group, Human Cancer Genetics Programme, Spanish National Cancer Research Center-CNIO, Madrid 28029, Spain; 3 Departament de Ciències Experimentals i de la Salut, Universitat Pompeu Fabra, Barcelona 08003, Spain

## Abstract

c-MYC oncogene is deregulated in most human tumours. Histone marks associated with transcriptionally active genes define high-affinity c-MYC targets. The mechanisms involved in their recognition by c-MYC are unknown. Here we report that c-MYC interacts with BPTF, a core subunit of the NURF chromatin-remodelling complex. BPTF is required for the activation of the full c-MYC transcriptional programme in fibroblasts. BPTF knockdown leads to decreased c-MYC recruitment to DNA and changes in chromatin accessibility. In *Bptf*-null MEFs, BPTF is necessary for c-MYC-driven proliferation, G1–S progression and replication stress, but not for c-MYC-driven apoptosis. Bioinformatics analyses unveil that BPTF levels correlate positively with c-MYC-driven transcriptional signatures. *In vivo*, *Bptf* inactivation in pre-neoplastic pancreatic acinar cells significantly delays tumour development and extends survival. Our findings uncover BPTF as a crucial c-MYC co-factor required for its biological activity and suggest that the BPTF-c-MYC axis is a potential therapeutic target in cancer.

## Abstract (web summary)

c-MYC genomic distribution is dictated by the epigenetic context but the mechanisms are unknown. Here, the authors show that c-MYC requires the chromatin reader BPTF to activate its transcriptional program and promote tumour development *in vivo*, suggesting that BPTF is a potential target for cancer therapy.

## Graphical abstract

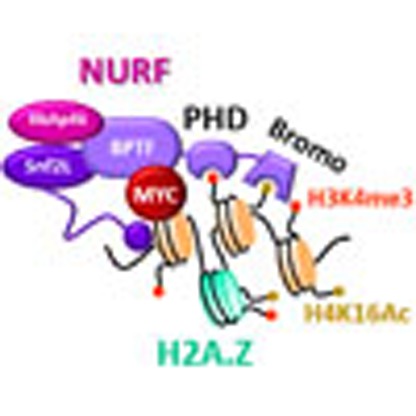

The *MYC* oncogenes encode a family of related sequence-specific bHLHZip transcription factors (c-, L- and N-MYC) that control a plethora of cellular functions including cell growth, proliferation, differentiation and apoptosis. c-MYC is deregulated in more than half of human cancers^1^, often in association with aggressive, poorly differentiated tumours^2^.

The oncogenic potential of c-MYC stems from its function as a transcriptional regulator that binds DNA on heterodimerization with MAX^3^. MYC-MAX heterodimers show a predilection for the palindromic ‘E-box' motif (CACGTG) found in regulatory elements of genes controlled by this complex^4^. Nonetheless, mounting evidence indicates that the genomic distribution of c-MYC-MAX complexes is influenced by factors other than sequence specificity, most notably the chromatin context^5^,^6^. High-affinity sites are bound by c-MYC in a wide variety of cell types and are typically enriched in CpG islands together with high levels of activating histone marks (H3K4me3, H3K79me, H3ac and H2A.Z). Low-affinity sites vary among cell types and are only engaged when c-MYC is expressed at high levels. Compared with high-affinity targets, they show a selective enrichment for macroH2A, H3K27me3 and H4K16ac^5^,^6^,^7^. Upon binding to its target promoters, c-MYC recruits multiple cofactors that affect the state of chromatin and the activity of RNA polymerases. Among them are chromatin-remodelling complexes (for example, SWI/SNF), acetyltransferases and methyltransferases that modify core histones (for example, P300/CBP-Associated Factor (PCAF)) and proteins associated with the basal transcriptional machinery (for example, P-TEFb)^8^,^9^,^10^.

The mechanisms involved in the recognition of the active chromatin configuration by c-MYC are poorly understood but likely involve the combined action of epigenetic ‘readers' and chromatin remodellers that modulate the accessibility of DNA in modified nucleosomes. A plausible candidate to act as a c-MYC tethering factor is NURF (ATP-dependent nucleosome-remodelling factor), an ISWI complex that uses ATP hydrolysis to catalyse nucleosome sliding^11^,^12^. Mammalian NURF consists of three subunits: BPTF, SNF2L and pRBAP46/48. BPTF (bromodomain PHD transcription factor) provides sequence specificity to NURF through interactions with transcription factors, histone variants and histone modifications of transcriptionally active genes (H3K4me3, H4K16Ac and H2A.Z)^12^,^13^,^14^. We found that BPTF is mutated in bladder tumours and its knockdown in cultured bladder cancer cells results in reduced proliferation^15^ and hypothesized that these effects might be mediated, in part, by c-MYC.

Here we show that BPTF and c-MYC are present in a protein complex. This interaction is critical for c-MYC function, since BPTF knockdown leads to a decrease in c-MYC binding to DNA, changes in chromatin accessibility and impaired activation of the c-MYC transcriptional programme. Consistent with this, BPTF expression in human tumours positively correlates with activation of c-MYC gene signatures. In addition, BPTF is necessary for the survival of c-MYC-overexpressing cells and for c-MYC-driven tumorigenesis in the mouse pancreas. These results highlight the potential of exploiting the BPTF-MYC axis in cancer therapy.

## Results

### BPTF depletion impairs c-MYC transcriptional activity

**Figure 1:**
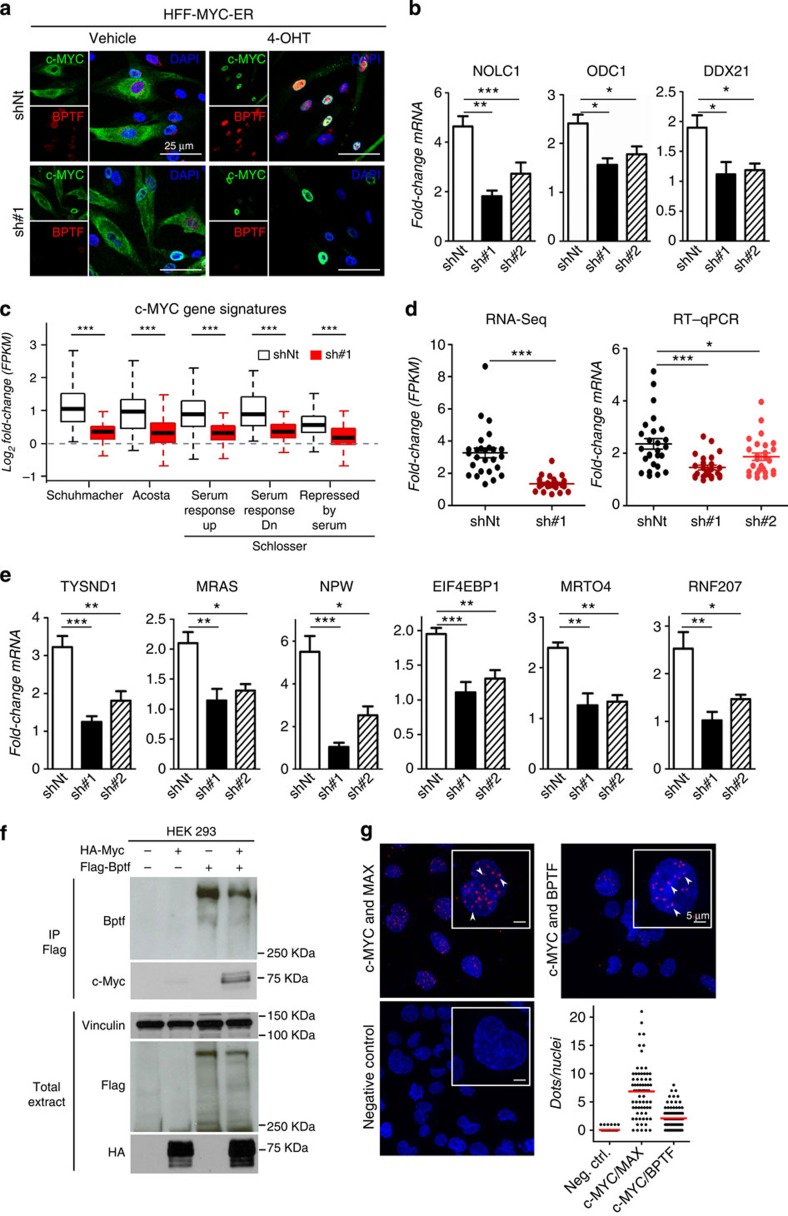
BPTF is required for c-MYC transcriptional activity. (**a**) Immunofluorescence staining of BPTF and c-MYC showing MYC-ER nuclear translocation upon 4-OHT treatment in control and BPTF-silenced HFF. Scale bar, 25 μm (**b**) Examples of expression of known c-MYC target genes, analysed by RT–qPCR, upon BPTF knockdown. Transcript levels were normalized against GAPDH and the vehicle-treated condition. Data are expressed as mean±s.e.m. (*n*≥3). *P* values were determined using an unpaired *t*-test. (**c**) Fold change in FPKM values of c-MYC-dependent gene sets enriched in 4-OHT-treated control cells. Values are displayed for both control and BPTF-silenced HFF MYC-ER cells. Gene sets tested and *P* values (Wilcoxon test): 1, Schuhmacher *et al.* ‘MYC targets up' (*P*=3.156 × 10^−15^); 2, Acosta *et al.* ‘proliferation independent MYC targets up' (*P*=2.034 × 10^−08^); 3, Schlosser *et al.* ‘MYC targets serum response up' (*P*=6.62 × 10^−09^); 4, Schlosser *et al.* ‘MYC targets serum response Dn' (*P*=6.11·10^−09^); 5, Schlosser *et al.* ‘MYC targets repressed by serum' (*P*=2.747·10^−14^). (**d**) Fold change in mRNA levels for the set of 20 genes used for validation. Left panel: data calculated from FPKM values. Right panel: data calculated from ≥3 independent experiments assessed by RT–qPCR. *P* values were determined using a Wilcoxon test. (**e**) Examples of genes included in the validation. Transcript levels were normalized against GAPDH and the vehicle-treated condition. Data are expressed as the mean±s.e.m. *P* value was determined using an unpaired *t*-test. (**f**) Co-immunoprecipitation of Flag-BPTF with HA-tagged c-MYC from lysates of transiently transfected 293T cells; western blotting with the indicated antibodies. (**g**) Endogenous BPTF and c-MYC interact in MIA PaCa-2 cells as shown by *in situ* proximity ligation assay. The interaction events are visible as red dots (nuclear staining in blue) and are marked by arrowheads. The interaction of MYC with MAX is shown as a positive control. Number of dots per nuclei was quantified manually (*n*=70 nuclei). Scale bar, 5 μm. *P<0.05; **P<0.01; ***P<0.001.

To assess whether BPTF is required for the transcriptional activity of c-MYC, human foreskin fibroblasts (HFFs) were stably transduced with the chimeric MYC-ER complementary DNA (cDNA; hereafter HFF MYC-ER) and infected with lentiviruses coding for either control (shNt) or BPTF-targeting short-hairpin RNAs (shRNAs; sh#1 and sh#2). To rule out proliferation-associated effects, and to avoid the interference of endogenous c-MYC, cells were driven to quiescence by serum starvation before treatment with 4-hydroxytamoxifen (4-OHT). Western blot and immunofluorescence analyses confirmed that the lentiviral shRNAs inhibited the expression of BPTF and did not interfere with either MYC-ER expression or nuclear translocation (Fig. 1a; Supplementary Fig. 1a). We examined the expression of a set of well-established c-MYC targets in control and BPTF-silenced HFF MYC-ER cells by real-time quantitative reverse transcriptase PCR (RT–qPCR). BPTF knockdown resulted in a significant impairment of the induction of 6/7 c-MYC messenger RNA (mRNA) targets with at least one of the two shRNAs (Fig. 1b; Supplementary Fig. 1b). To extend these findings, we used *Bptf*-null mouse embryonic fibroblasts (MEFs)^16^ transduced with MYC-ER. Successful recombination of the floxed allele was demonstrated by PCR and RT–qPCR analysis (Supplementary Fig. 1c,d). In these cells, addition of 4-OHT also resulted in an impaired activation of four c-MYC targets tested^17^ (Supplementary Fig. 1e).

To further investigate the hypothesis that BPTF is required for c-MYC-dependent transcription, we performed global RNA profiling (RNA-Seq) using HFF MYC-ER cells. Normalization with spike-in standards^18^ yielded ∼6,700 differentially expressed genes in 4-OHT-treated control cells relative to vehicle (Supplementary Data 1). Gene Set Enrichment Analysis (GSEA) revealed that the genes upregulated upon 4-OHT addition were significantly enriched in c-MYC-dependent transcriptional signatures^19^,^20^,^21^ and gene ontology pathways classically associated with c-MYC function (that is, ribosome biogenesis and translation, mitochondrial function, and RNA/ribosomal RNA/transfer RNA processing). Moreover, genes downregulated in control 4-OHT-treated cells overlapped with gene sets known to be repressed by c-MYC^22^,^23^ (Supplementary Fig. 1f; Supplementary Data 2). These findings are consistent with the specific transcriptional regulation of the c-MYC signature and validate the approach used.

BPTF knockdown in HFF MYC-ER cells resulted in a reduced transcriptional response to 4-OHT, both up- and downregulated genes being significantly affected (Supplementary Fig. 1g). The mechanisms involved in c-MYC-mediated repression have not been fully elucidated; therefore, we focused on its best-established role as a transcriptional activator^18^. We found an impaired activation of five independent c-MYC signatures in BPTF-silenced cells (Fig. 1c). These results were validated by RT–qPCR for an additional 20 genes, 19 of which are c-MYC chromatin immunoprecipitation sequencing (ChIP-Seq) targets in at least one cell line profiled by ENCODE (Supplementary Data 1 and 3). The extent of induction of these genes was significantly reduced in HFF MYC-ER cells transduced with both BPTF-targeting shRNA lentiviruses (average fold change (4-OHT versus vehicle) for shNt, 2.44; sh#1, 1.52; sh#2, 1.97; shNt versus sh#1, *P*<0.001; shNt versus sh#2, *P*=0.049; unpaired *t*-test; Fig. 1d). Representative results are shown in Fig. 1e. These data indicate that BPTF is required for c-MYC-mediated gene regulation.

### BPTF interacts with c-MYC

To activate transcription, c-MYC recruits chromatin-modifying and -remodelling complexes that hyperacetylate histones, displace nucleosomes and increase DNA accessibility at target regions^8^,^24^,^25^. BPTF recognizes histone marks present in both high- and low-affinity c-MYC target promoters and is involved in chromatin remodelling. We thus reasoned that BPTF association with c-MYC might mechanistically explain the suppression on c-MYC transcription. Immunoprecipitation of Flag-BPTF followed by western blotting to detect HA-MYC from transfected 293T lysates revealed both proteins in the same complex (Fig. 1f). The interaction between endogenous c-MYC and BPTF was validated in MIA PaCa-2 pancreas cancer cells, expressing high levels of both proteins, using the *in situ* proximity ligation assay (isPLA) and affinity-purified rabbit antibodies recognizing BPTF residues 913–942 (Fig. 1g; Supplementary Fig. 2a,b). Together, these data suggest that BPTF interacts with c-MYC.

### BPTF regulates c-MYC binding to DNA

The E-box motif recognized by c-MYC is very widely represented at the genome-wide level and the findings described above suggested that BPTF serves to recruit c-MYC to its target promoters through its ability to recognize specific histone marks. To test this hypothesis, we conducted ChIP with anti-c-MYC antibodies followed by massive parallel sequencing (ChIP-Seq) in HFF MYC-ER cells (Supplementary Data 4). A total of 1,397 peaks were identified in 4-OHT-treated control cells. In agreement with previous reports, the analysis of the density profiles of the distance between the summit of peaks and transcription start sites (TSSs) showed that c-MYC-binding sites concentrate around the TSS (Supplementary Fig. 3a)^5^,^26^. ChIP peaks occurred within promoter regions (TSS±3 kb; 35.6%), gene bodies (intragenic; 25%) and further upstream or downstream (intergenic; 39.4%). Sequence analysis of c-MYC-bound regions with MEME^27^ unveiled a significant over-representation of the MYC-MAX-binding motif (*P=*2.8 × 10^−69^; Supplementary Fig. 3b). Moreover, GSEA analysis of c-MYC-bound promoters showed highly statistically significant overlap with three transcriptional signatures of c-MYC-dependent genes and with biological modules associated with c-MYC function (for example, cell proliferation; Supplementary Fig. 3c; Supplementary Data 5). Furthermore, genes directly bound by c-MYC showed a significantly higher mRNA induction than those lacking a ChIP-Seq peak (Supplementary Fig. 3d).

**Figure 2:**
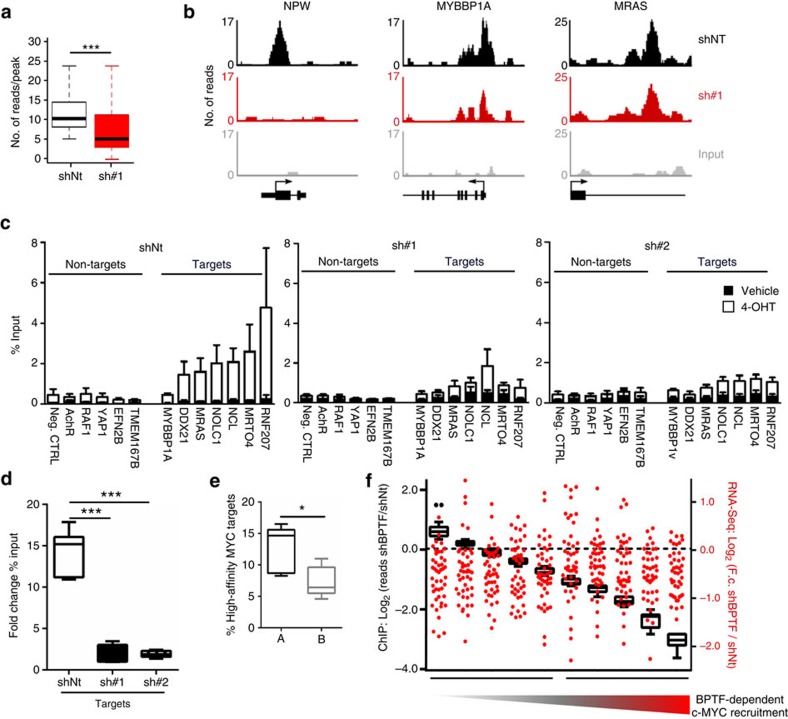
BPTF silencing interferes with c-MYC recruitment to its target genes. (**a**) Box plot showing the intensity of c-MYC ChIP-Seq signal (reads per peak) at MYC-enriched regions in control and BPTF-silenced HFF MYC-ER cells. MYC-enriched regions were defined in 4-OHT-treated control cells. c-MYC-binding intensity was measured as number of reads per peak. ***P<0.001 (Wilcoxon test). (**b**) Representative snapshots of c-MYC-bound genomic regions in control and BPTF-silenced HFF MYC-ER cells after stimulation with 4-OHT. (**c**) ChIP analysis of c-MYC enrichment at the promoters of ‘target' and ‘non-target' genes in control and BPTF-silenced HFF MYC-ER cells in the presence (white) or absence of 4-OHT (black). ChIP values are expressed as average±s.e.m. of % input chromatin. An isotype-matched IgG antibody was used as control (Supplementary Fig. 3f) and ≥3 independent experiments were analysed per promoter region. (**d**) Fold change in % of input following 4-OHT addition, averaged for the two different promoter populations in control and BPTF-silenced cells. ***P<0.001 (unpaired *t*-test). (**e**) Groups A and B are defined as in **f**. High-affinity MYC targets are significantly enriched among the genes for which MYC recruitment is less affected by BPTF knockdown. *P<0.05 (unpaired *t*-test). (**f**) c-MYC target genes ranked according to the change in c-MYC binding at their promoters after BPTF silencing. BPTF dependency of c-MYC recruitment to DNA is calculated as the Log_2_ (reads shBPTF/reads shNt; left *y* axis). For the same collection of ranked genes, the transcriptional response to 4-OHT is shown (scatter plot right *y* axis). BPTF dependency of 4-OHT-dependent mRNA induction is calculated as the Log_2_ (F.c. shBPTF/F.c. shNt). Four data points are outside the right *y* axis limits.

To determine whether BPTF silencing interfered with c-MYC recruitment to chromatin, we analysed the magnitude and distribution of c-MYC ChIP-Seq peaks upon BPTF knockdown. Globally, c-MYC-binding intensity was significantly lower in shBPTF-expressing cells (*P*<2.2 × 10^−16^, Wilcoxon test) (Fig. 2a,b). This reduction was not uniformly distributed at the genome-wide level: only 50.2% of the peaks showed a read number fold change ≥2 and this was unrelated to peak intensity, suggesting that the differences did not result from an inefficient ChIP. These results indicate that BPTF silencing selectively affects a subset of c-MYC ChIP-Seq peaks. We validated these observations by ChIP–qPCR on gene promoters for which a peak was identified in the ChIP-Seq experiment (‘target'), as well as on ‘non-target' control genomic regions (Supplementary Data 3 and 6), in at least three independent experiments. By this approach, c-MYC recruitment to target genes was significantly reduced in cells infected with the BPTF-targeting shRNAs (Fig. 2c,d; Supplementary Fig. 3e).

c-MYC target regions cluster into ‘high-affinity' or ‘low-affinity' sites. We investigated the effect of BPTF knockdown on c-MYC recruitment to both types of targets, as previously defined^5^,^26^, and observed a significant enrichment of high-affinity sites among the genes less affected by BPTF silencing (Fig. 2e,f). These data suggest that BPTF requirement for target recognition by c-MYC depends on the epigenetic context: while dispensable for c-MYC binding to H3K4me3-rich ‘high-affinity' promoters, it is necessary for c-MYC binding to low-affinity sequences, possibly through the recognition of H4K16ac. The effect of BPTF silencing on the induction of c-MYC target mRNAs was independent from the extent of reduction in c-MYC binding at their promoters (Fig. 2f; Supplementary Data 7), suggesting that c-MYC requires BPTF to activate transcription at low-affinity targets genes.

### BPTF is required for c-MYC-induced chromatin remodelling

**Figure 3:**
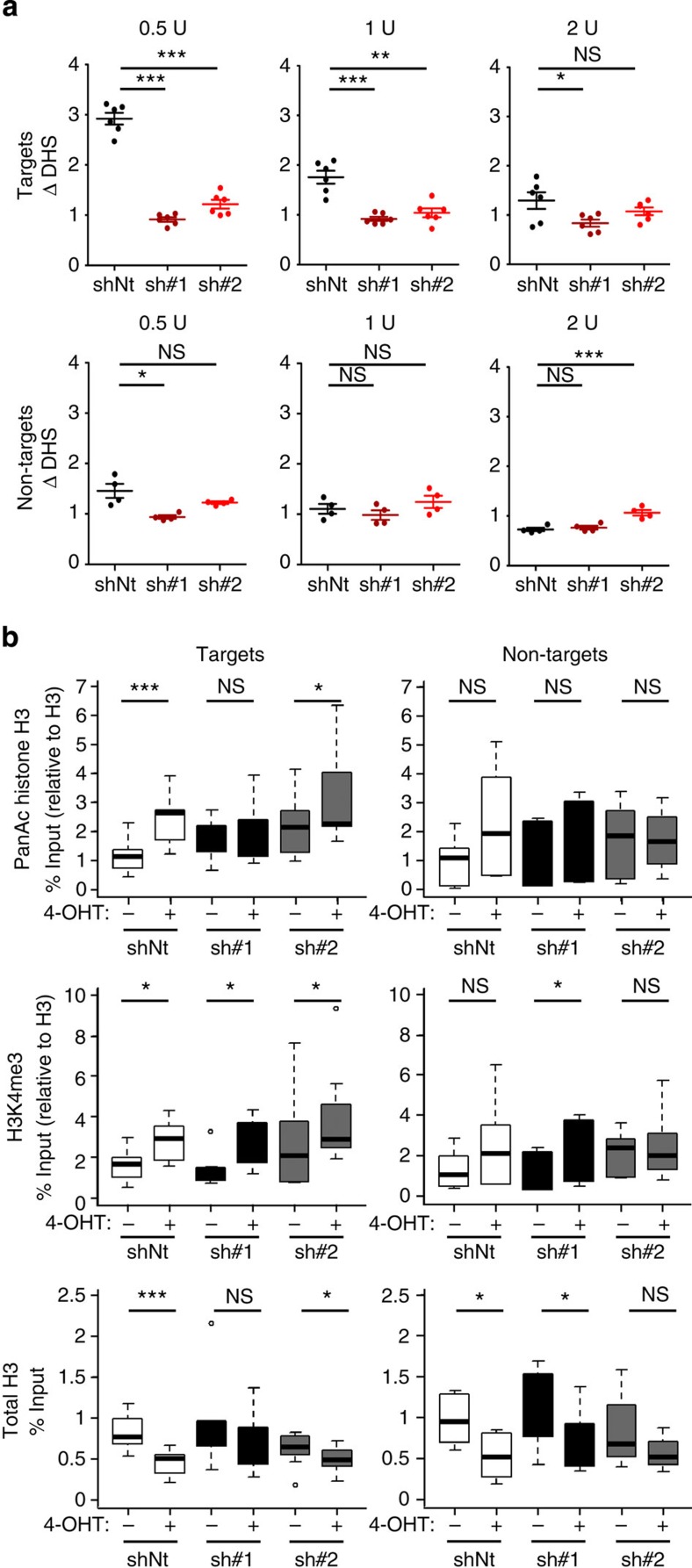
BPTF is required for MYC-induced chromatin remodelling. (**a**) DNase I hypersensitivity (DHS) at MYC-bound regions in control and BPTF-silenced HFF MYC-ER cells, determined by enzyme titration. Dots represent the average values of seven independent experiments. *P* values were determined using paired *t*-test. (**b**) ChIP analysis of Pan AcH3, H3K4me3 and total H3 levels at the promoter of ‘target' and ‘non-target' genes in control and BPTF-silenced HFF MYC-ER cells. Each gene was assayed in at least three independent experiments. ChIP values are expressed as % of input and normalized for total histone H3 (with the exception of total H3). *P* values were determined using paired *t*-test. *P<0.05; **P<0.01; ***P<0.001.

To assess whether BPTF knockdown led to changes in DNA accessibility, we performed quantitative DNAse I hypersensitivity assays, as described earlier^28^. DNAse I hypersensitive sites mark *cis*-regulatory elements (that is, enhancers or promoters) and result from the cooperative binding of transcription factors and chromatin-remodelling complexes^29^. We analysed the DNA accessibility of c-MYC ‘target' promoters validated in Fig. 2c, in the presence or absence of 4-OHT, and included ‘non-target' regions for comparison. 4-OHT addition to HFF MYC-ER cells led to an increased sensitivity to DNAse I of ‘target' regions in control (*P*<0.001, paired *t*-test) but not in BPTF-silenced cells (Fig. 3a (top); Supplementary Data 8). There was no consistent effect on ‘non-target' promoters (Fig. 3a (bottom) and Supplementary Data 8). Overall, these results indicate that attenuation of the c-MYC transcriptional response resulting from BPTF knockdown is associated with changes in DNA accessibility, suggesting that BPTF is necessary for the c-MYC-induced remodelling of target chromatin. We next analysed the levels of acetylated H3 and H3K4me3 in ‘target' and ‘non-target' promoters by ChIP–qPCR. As reported previously, c-MYC activation in control cells was associated with the selective hyperacetylation of histone H3 in ‘target' promoters (*P*=0.002, paired *t*-test). Importantly, this effect was lost upon BPTF knockdown (Fig. 3b, top). By contrast, the levels of H3K4me3 were unaffected by BPTF silencing (Fig. 3b, middle). c-MYC activation in control cells was associated with a significant decrease in total H3 at c-MYC target promoters, suggesting chromatin remodelling. BPTF silencing did not alter the basal levels of H3 at these sites but it impaired H3 depletion (Fig. 3b, bottom), in agreement with the DNAse I hypersensitivity data. In summary, these results indicate that BPTF regulates chromatin accessibility at c-MYC target promoters.

### BPTF is required for a subset of c-MYC biological functions

MYC proteins regulate a wide variety of biological processes including cell proliferation, differentiation and apoptosis. Physiological c-MYC levels induce DNA synthesis through the transcriptional activation of cell cycle-related genes^30^ and by modulating the activity of DNA replication origins^31^. Here we analyse the requirement of BPTF for c-MYC-induced proliferation, replication stress and apoptosis.

**Figure 4:**
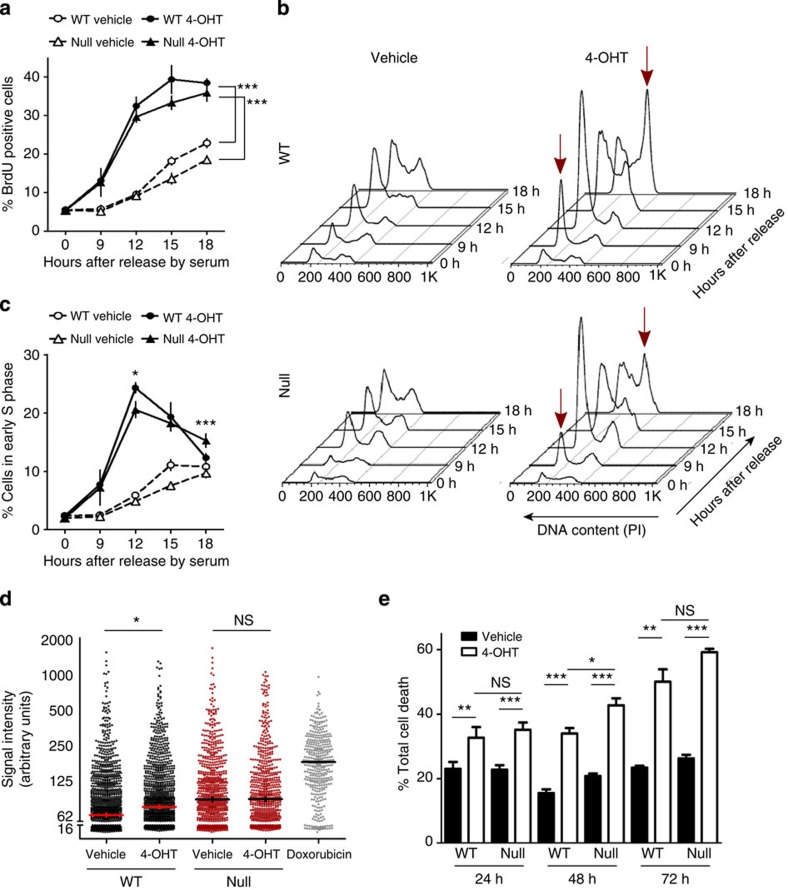
Bptf is required for MYC-induced proliferation of MEFs and replication stress but not for apoptosis. (**a**) WT (*n*=4) and *Bpft*-null (*n*=4) MEFs transduced with MYC-ER were seeded at high density, arrested with 0.5% FBS for 48 h and stimulated with serum in the presence/absence of 4-OHT. At the indicated time points, cells were pulse labelled with BrdU for 1 h before collected. Data are represented as mean±s.e.m. ***P<0.001 (paired *t*-test). (**b**) Histograms depicting the ploidy of BrdU-positive cells throughout the experiment described in **a**. (**c**) Quantification of early S-phase cells. The rate of loss of BrdU^+^ early S-phase cells represents S-phase progression. Data are represented as mean±s.e.m. *P<0.05; ***P<0.001 (unpaired *t*-test). (**d**) Replication stress—intensity of γH2AX signal in WT and Bpft-null MYC-ER MEFs (*n*=3 per group) in the presence or absence of 4-OHT for 48 h. Doxorubicin-treated cells were used as control. *P<0.05 (paired *t*-test) (**e**) WT (*n*≤4) and *Bpft*-null (*n*≤4) MEFs expressing MYC-ER were seeded at high density and then transferred to 0.5% FBS with or without 4-OHT (2μM). Apoptosis was measured as the proportion of Annexin V-postivive cells at the indicated time points. *P<0.05; **P<0.01; ***P<0.001 (paired *t*-test to compare vehicle versus 4-OHT; unpaired *t*-test to compare WT versus *Bptf*-null cells).

To determine whether BPTF is required for c-MYC-induced proliferation, we used wild-type (WT) and *Bptf*-null MEFs. Cells were co-infected with lentiviruses coding for Cre recombinase and the MYC-ER fusion protein. Quiescent MEFs were induced to re-enter the cell cycle by adding fetal bovine serum (FBS)±4-OHT and S-phase entry was assessed by 5-bromodeoxyuridine (BrdU) uptake. 4-OHT-treated WT and *Bptf*-null cells showed a significantly higher percentage of cells in S-phase than vehicle-treated cells as early as 9 h after FBS+4-OHT stimulation (*P*=0.014, paired *t*-test), indicative of MYC-induced G1/S progression (Fig. 4a). DNA content analysis of BrdU^+^ cells followed by quantification of cells in early S-phase showed that BPTF deletion resulted in a significantly delayed progression through S-phase in MYC-ER-activated cells (at 18 h, *P*<0.001, unpaired *t*-test). There were no effects on vehicle-treated cells (Fig. 4b,c).

c-MYC overexpression and/or deregulation is associated with unscheduled firing of DNA replication origins, DNA damage response and checkpoint activation^32^. In WT MYC-ER MEFs, 4-OHT addition led to an increase in the median signal intensity of pan-nuclear γH2AX (*P*=0.017, paired *t*-test), indicative of replication stress. In *Bptf*-null cells, a slight increase was observed in basal conditions but there was no significant change upon addition of 4-OHT (Fig. 4d).

c-MYC can induce apoptosis when expressed from an ectopic promoter in the presence of limiting survival signals or upon stress^33^. To assess whether BPTF is required for MYC-induced apoptosis, WT and *Bptf*-null MYC-ER MEFs were seeded at high density and cultured in 0.5% FBS containing either vehicle or 4-OHT. Apoptosis was quantified by Annexin V staining and 4,6-diamidino-2-phenylindole (DAPI) exclusion. MYC-ER activation triggered a robust apoptotic response both in WT and in *Bptf*-null MEFs (Fig. 4e), indicating that BPTF is differentially required for a subset of c-MYC biological functions.

### Deregulation of the c-MYC:BPTF axis in human cancer

**Figure 5:**
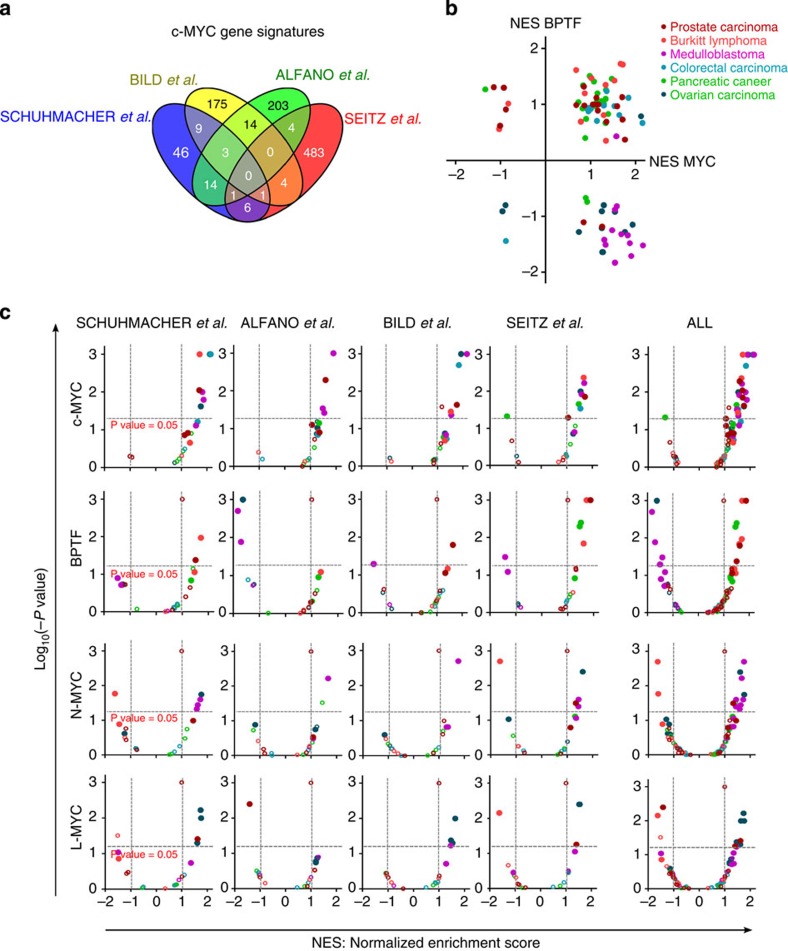
BPTF expression correlates with c-MYC signatures in human tumours. (**a**) Venn diagram showing the overlap of the c-MYC signatures used in the following analyses. (**b**) Dot plot of normalized enrichment scores (NES) of the four c-MYC signatures based on GSEA. NES values were calculated for each data set previously ranked-ordered by either BPTF or c-MYC levels. (**c**) Volcano plots of NES and enrichment *P* values of c-MYC signatures based on GSEA. NES values were calculated for each data set previously ranked-ordered by BPTF, c-MYC, N-MYC or L-MYC mRNA levels. Filled circles represent gene sets with a false discovery rate <0.25.

To gain insight into the role of the c-MYC:BPTF axis in human cancer, we analysed public omics data sets. First, we compared BPTF and c-MYC levels with the activation of c-MYC signatures in a collection of 20 expression data sets from Oncomine and Gene Expression Omnibus (GEO) encompassing human tumours of diverse origin (Supplementary Data 9) driven by different MYC family members: Burkitt lymphoma (BL), colorectal, prostate and pancreatic tumours are mainly driven by c-MYC^34^,^35^,^36^, whereas medulloblastoma and ovarian carcinoma commonly show amplification and/or overexpression of N-MYC and L-MYC, respectively^37^,^38^. BPTF is overexpressed in tumours together with c-MYC and, in some cases, N-MYC and L-MYC (Supplementary Fig. 4a). Samples within each data set were rank-ordered by the mRNA levels of either BPTF, c-MYC, N-MYC or L-MYC and then interrogated by single-sample GSEA (ssGSEA)^39^ for enrichment of four c-MYC gene sets showing a modest degree of overlap (Fig. 5a). c-MYC signatures correlated with c-MYC expression levels in BL, colorectal, prostate and pancreatic carcinomas. In these tumours, BPTF expression levels also correlated positively with c-MYC signatures (Fig. 5b,c). By contrast, N-MYC and L-MYC expression levels correlated with c-MYC expression signatures only in medulloblastoma and ovarian carcinoma, respectively, and in these tumours the signatures correlated negatively with BPTF expression levels (Fig. 5b,c). These data suggest that BPTF is more selectively associated with the biological activity of c-MYC than with that of N-MYC or L-MYC.

**Figure 6:**
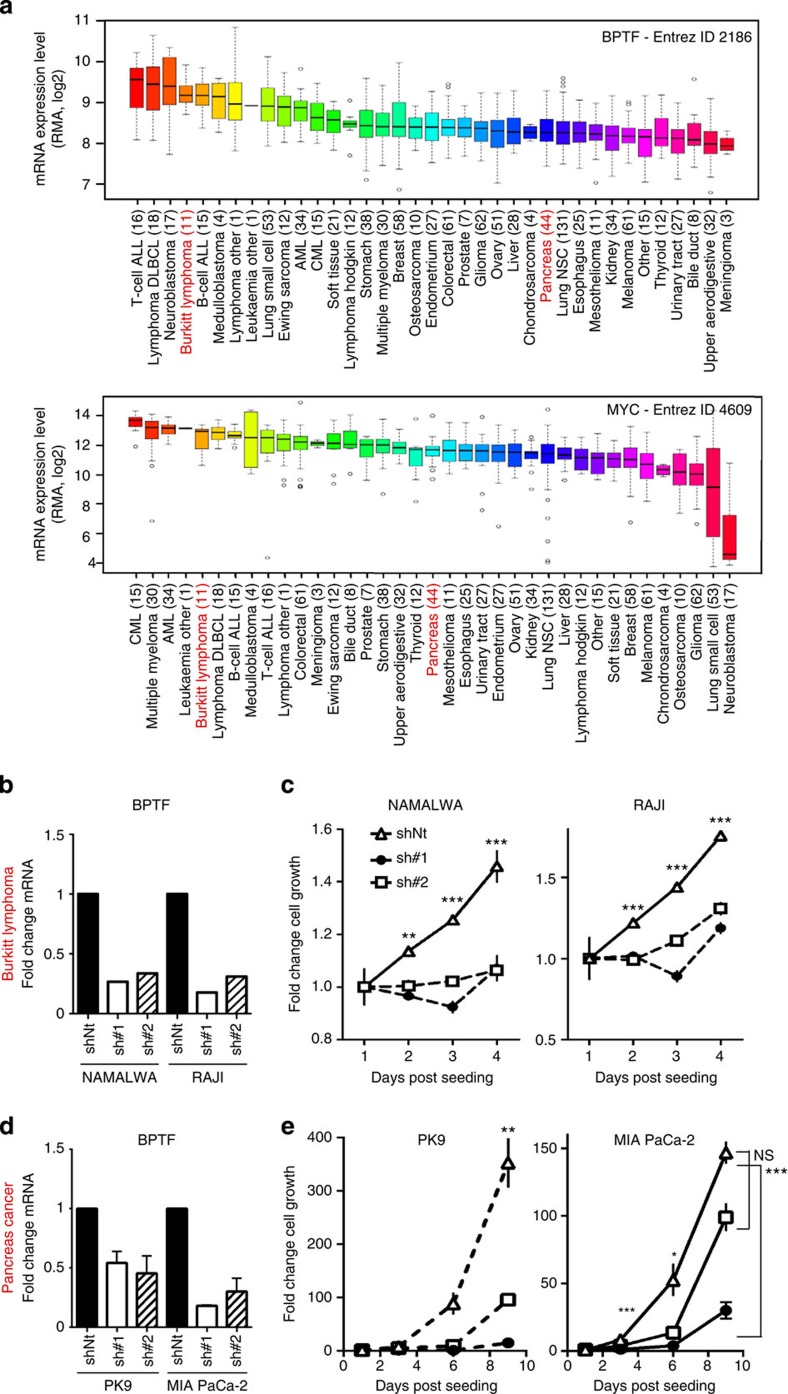
BPTF knockdown suppresses the proliferation of BL and pancreatic cancer cells. (**a**) Top: box plot showing the relative BPTF mRNA levels across the different tumour types, extracted from CCLE_Expression_Entrez_ID_2186, with gene-centric robust multiarray analysis-normalized mRNA expression data. Bottom: box plot showing the relative c-MYC mRNA levels across a panel of human cell lines, extracted from CCLE_Expression_Entrez_ID_4609. The number of cell lines of each tumour type analysed is indicated in parentheses. Effective knockdown of BPTF in BL cells (**b**) and pancreatic cancer cells (**d**), assessed by RT–qPCR. Transcript levels were normalized against GAPDH and the sh-control samples. Proliferation analysis of two BL cell lines (NAMALWA and RAJI; **c**) and two pancreatic cancer cell lines (**d**) transduced with either control (shNt) or BPTF-targeting shRNAs (sh#1 and #2) (*n*=5). *P<0.05; **P<0.01; ***P<0.001.

On the basis of these findings, we focused on tumour types associated with c-MYC hyperactivation. First, we analysed the expression of BPTF and c-MYC in the Cancer Cell Line Encyclopedia data set. As shown in Fig. 6a, expression of both genes was positively correlated across all tumour types. We then selected BL and pancreatic ductal adenocarcinoma (PDAC) cells for further analysis. BL is a paradigm of a c-MYC-addicted human tumour resulting from chromosomal translocations placing c-*MYC* under the control of Ig regulatory sequences leading to c-MYC overexpression^34^. Consistently, BL-derived lines are among the highest expressors of both BPTF and c-MYC. PDAC-derived cell lines express intermediate levels of both genes (Fig. 6a) and their dependency from c-MYC is generally not the result of primary genetic alterations in this gene. BPTF knockdown in NAMALWA and RAJI BL cells was accompanied by reduced cell proliferation (Fig. 6b,c). Similarly, BPTF knockdown in PK9 and MIA Paca-2 cells led to reduced proliferation (Fig. 6d,e), indicating that tumour types with different c-MYC dependency require BPTF to sustain proliferation.

### BPTF inactivation prolongs the survival of *Ela1-Myc* mice

**Figure 7:**
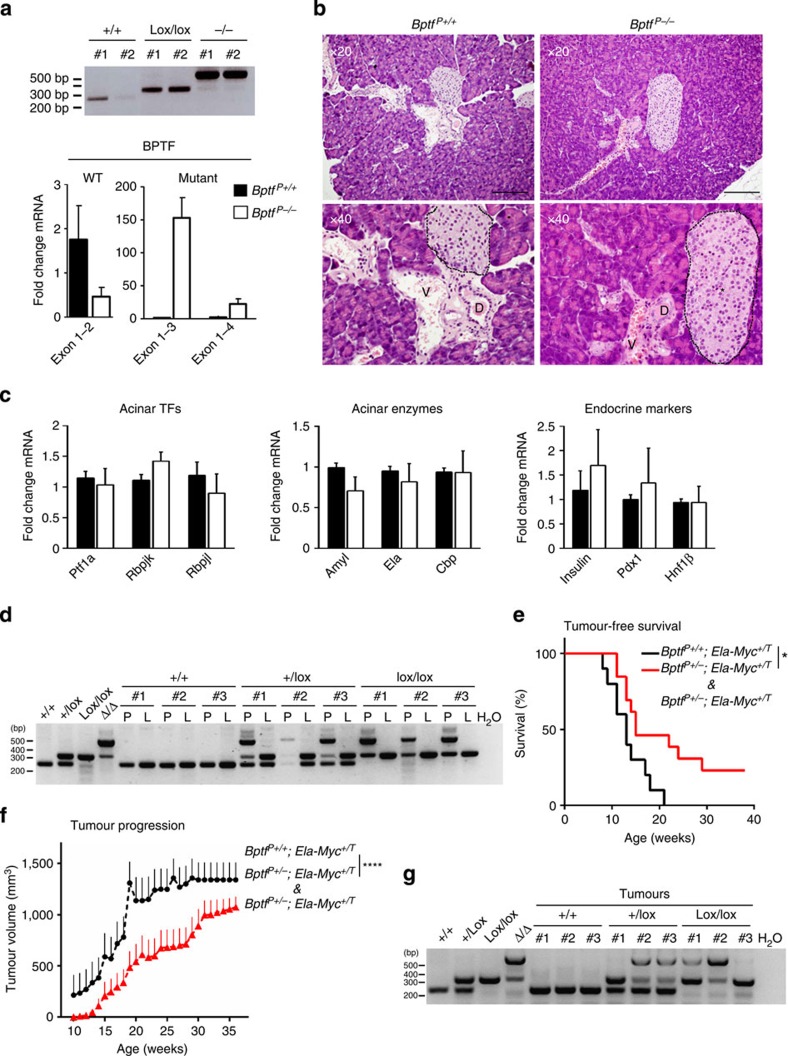
BPTF is dispensable for pancreatic differentiation but its inactivation delays the onset and progression of c-MYC-driven pancreatic tumors. (**a**) PCR on genomic DNA showing efficient recombination at the *Bptf* locus in *Bptf*^P−/−^ pancreas (top). RT–qPCR analysis of BPTF WT and mutant mRNA species in control (*n*=6) and *Bptf*^*P−/−*^ (*n*=7) mice (bottom). Transcript levels were normalized against actin and the WT condition. (**b**) Haematoxylin–eosin staining of WT and *Bptf*^P−/−^ mouse pancreatic sections. D, duct; V, blood vessel; *, islet of langerhans. Scale bar, 200 μm. (**c**) mRNA expression of acinar transcription factors, digestive enzymes and endocrine markers in pancreata of WT and *Bptf*^*P−/−*^ mice assessed by RT–qPCR (*n*=3 per genotype). Transcript levels were normalized against actin and the WT condition. (**d**) PCR on pancreas (P) genomic DNA assessing the extent of recombination at the *Bptf* locus in 5–7-week-old *Ptf1a-CreERT2*^*+/KI*^*;Ela-Myc* mice of the corresponding genotypes. Liver (L) samples were used as negative controls. (**e**) Kaplan–Meier curves of tumour-free survival are shown for *Ela-Myc* mice of the indicated *Bptf* genotypes. *P<0.05 (log-rank test). (**f**) Tumour volume of *Ela-Myc* mice of the indicated *Bptf* genotypes as determined by ultrasound. Data are expressed as the mean±s.e.m. ****P<0.00001 (Wilcoxon test). (**g**) PCR analysis of genomic DNA from tumours arising in *Bptf*^*P+/+*^, *Bptf*^*P−/+*^ and *Bptf*^*P−/−*^; *Ela-Myc* mice.

On the basis of the important role of c-MYC in cancer, the bioinformatics analyses, and results of knockdown experiments shown above, we asked whether BPTF is also required for the oncogenic effects of c-MYC *in vivo* using genetic mouse models. Because there is increasing evidence that differentiation acts as a tumour suppressor mechanism in the pancreas^40^,^41^, we first analysed whether *Bptf* deletion in this tissue results in altered cell differentiation. We analysed the pancreas of 8-week-old mice in which *Bptf* was deleted at E9.5–E10.5 using *Ptf1a-Cre* as a deletor strain. Very efficient recombination and downregulation of the WT Bptf transcript was achieved (Fig. 7a). The pancreas of *Bptf*^*−lox/lox*^;*Ptf1a*^*Cre*^ mice was histologically normal and RT–qPCR analysis of acinar and endocrine markers failed to reveal evidence of altered differentiation (Fig. 7b,c).

To determine the effects of *Bptf* deletion on tumour development/progression, we used *Ela1-Myc* mice where c-MYC overexpression is driven by the acinar-specific *Elastase 1* promoter. These mice develop highly aggressive acinar and ductal pancreatic tumours with a 100% penetrance^42^. We generated *Bptf*^*lox/lox*^*;Ptf1a-CreERT2*^*+/KI*^*;Ela-Myc* mice (*Bptf*^*P−/−*^*;Ela-Myc*) where BPTF can be depleted in PTF1A^+^ acinar cells at will. Tamoxifen was administered orally to 5–7-week-old mice, when dysplastic acinar cells are already detectable. Oral administration of tamoxifen resulted in recombination of 60–70% of the *Bptf* alleles as assessed by PCR on genomic DNA (Fig. 7d). A cohort of *Bptf*^*P+/+*^*;Ela1-Myc* (*n*=10), *Bptf*^*P+/−*^*;Ela1-Myc* (*n*=5) and *Bptf*^*P−/−*^*;Ela1-Myc* (*n*=8) mice was monitored weekly by ultrasound to determine tumour-free survival and animals were killed when tumour burden reached ethical end points or mice showed overt signs of morbidity. *Bptf*^*P+/+*^*;Ela1-Myc* mice displayed the expected course of pancreatic cancer onset, with a 50% disease-free survival of 13 weeks^42^,^43^. In contrast, *Bptf*^*P+/−*^*;Ela1-Myc* and *Bptf*^*P−/−*^*;Ela1-Myc* mice showed significantly delayed tumour onset and a correspondingly delayed tumour progression (Fig. 7e,f). To further understand the molecular basis of tumorigenesis in these mice, we analysed tumour tissues obtained at the time of death and evaluated by PCR the extent of recombination at the *Bptf* locus: we found evidence of tumour progression in both *Bptf*-deleted cells and in escaper cells in which *Bptf* had not been deleted (Fig. 7g). In summary, these data indicate that BPTF is necessary for the initiation and maintenance of c-MYC-driven pancreatic tumours.

## Discussion

The broad range of biological activities of c-MYC, its involvement in a wide variety of human tumours, and its unique ability to regulate a large fraction of the genome have spurred a great interest in the study of the molecular mechanisms involved in the c-MYC-mediated control of gene expression.

A prominent feature of c-MYC is that sequence-specific DNA binding is only one of the requirements for its regulatory activity. Its genomic distribution is influenced by the epigenetic context, which is determined by histone modifications, histone variants and CpG island distribution^5^,^6^. However, how these features relate to c-MYC engagement to promoters is not known. Here we provide evidence that BPTF interacts with c-MYC and is a key determinant of its genomic distribution and biological activity.

c-MYC target promoters can be classified according to their binding affinity and histone marks. High-affinity sites display high levels of H3K4me3, H2A.Z and unmethylated CpGs. Low-affinity sites are enriched in repressive histone marks (for example, H3K9me) together with H4K16Ac and are only occupied when c-MYC levels are high^5^,^6^. BPTF can recognize histone marks in both types of promoters; however, low-affinity promoters are more sensitive to BPTF levels. These results support the hypothesis that protein–protein interactions between c-MYC and other chromatin-bound complexes are critical to target recognition by c-MYC^44^. BPTF binds H3K4me3 through its PHD domains^45^ and its knockdown does not affect the levels of this histone mark at c-MYC target promoters. By contrast, BPTF silencing reduces the hyperacetylation of H3, commonly associated to c-MYC activation^46^. These data suggest that BPTF, either on its own or through its association with c-MYC, is required to recruit and/or modulate the activity of histone acetyltransferases (HATs) or histone deacetylases at promoters. Precedents for this kind of regulation are found in *Drosophila melanogaster*, where Nurf301 is needed for the histone acetyl-transferase ATAC to access chromatin and maintain the decondensed architecture of the male X chromosome^47^. More work is required to determine how BPTF regulates the recruitment of HATs/histone deacetylases by c-MYC.

BPTF silencing not only affected the transcriptional response of promoters where c-MYC binding was reduced but also of those where recruitment was unaffected. We propose that this results from defective chromatin remodelling, as suggested by the fact that BPTF silencing blocks the increase in DNA accessibility at c-MYC promoters typically linked to c-MYC activation. c-MYC recruits the chromatin remodeller SWI/SNF^8^, whose activity is partially inhibited by the linker histone H1 (ref. 48). NURF is necessary for H1 displacement at the promoters of progesterone receptor target genes^13^. Thus, we hypothesize that NURF-mediated eviction of H1 is a pre-requisite for subsequent remodelling and nucleosome eviction by SWI/SNF. Moreover, BPTF may either prevent or enhance the binding of transcription factors to DNA sequences adjacent to CCCTC-Binding Factor (CCTC)-binding sites. For example, the binding of KLF4 in the vicinity of CTCF sites is prevented by BPTF due to its nucleosome-remodelling activity^49^.

The recognition of active histone marks in regions of open chromatin by BPTF may facilitate and stabilize c-MYC binding. However, alternative scenarios are possible and the mechanisms through which c-MYC co-factors operate may differ at high- and low-affinity promoters. Our studies do not allow us to conclude on the sequence of recruitment of BPTF and c-MYC to target promoters. This is, in part, due to the fact that the use of RNA interference leads only to a partial reduction of BPTF levels. Therefore, it is possible that—when present at low levels—BPTF could differentially bind to histone marks and thereby affect gene expression.

Recently, WDR5 and c-MYC have been shown to interact directly. A c-MYC-WBM mutant unable to bind WDR5 showed reduced chromatin recruitment capacity, suggesting that WDR5 is important for c-MYC binding to chromatin^10^. A direct comparison of BPTF and WDR5 is required to determine the specific role of each of these proteins in the regulation of c-MYC activity.

The impact of BPTF silencing on c-MYC recruitment to distal enhancer elements remains to be determined. Active enhancers are characterized by high H3K4me1–2, H3K27ac, recruitment of the HAT p300 and the presence of transcription factor-binding motifs and DNAse I hypersensitivity sites^50^,^51^,^52^. The C-terminal PHD finger of BPTF binds H3K4me2 (ref. 53). It is thus possible that NURF is recruited to enhancers through H3K4me2, along with other chromatin remodellers such as CHD7 and BRG1 (refs 54, 55). Furthermore, as indicated above, BPTF interacts with CTCF that can bind and regulate nucleosome distribution at enhancers^49^.

c-MYC has a wide range of biological activities determined, in part, by its expression levels. Therefore, we asked for which of them is BPTF required. In multiple cell types, we show that BPTF is essential for c-MYC-driven proliferation. However, it is not necessary for MYC-triggered apoptosis in MEFs. Omomyc, a c-MYC mutant protein that cannot dimerize with MAX and does not bind E-boxes, also displays selective effects on c-MYC function: it interferes with proliferation but it exacerbates apoptosis triggered by high c-MYC levels^56^,^57^. The selective effect of BPTF knockdown on a subset of c-MYC functions could be related to the mechanisms of transcriptional activation of the genes involved. To induce cell proliferation, c-MYC binds directly the promoter of genes participating in DNA replication and cell cycle control (that is, MCM5, MCM6 or DBF4) and enhances their transcription^26^. By contrast, c-MYC-driven apoptosis is mostly indirect, through pathways that do not require binding to DNA, and involves the stabilization of p19^ARF^ and p53 or the downregulation of anti-apoptotic BCL-2 through inhibition of MIZ-1 (ref. 58). We therefore propose that BPTF is only required for those c-MYC functions involving direct binding to chromatin.

*MYC* oncogenes have partially overlapping biological functions but display distinct tissue-specific expression and transforming activity^59^. c-MYC and N-MYC can functionally replace one another in certain contexts (for example, murine development) and share similar oncogenic potency^60^,^61^, but N-MYC is distinctly involved in the regulation of genes involved in neural development^62^. Despite that less is known on L-MYC, it appears to differ from the other family members^22^. Our genomic analyses suggest that BPTF may differentially affect the oncogenic activity of MYC family members and BPTF levels correlate positively with a c-MYC transcriptional signature. CTCF is required for c-MYC expression^63^; it is possible that BPTF and CTCF interact to activate c-MYC expression through the recruitment of transcription factors to its promoter. More work is required to determine the relevance of the epigenetic context of, and the requirement of BPTF for, the activity of N-MYC and L-MYC.

In recent years, genetic mouse models have shown that tumours become addicted to c-MYC^64^. In inducible c-MYC-driven models, established tumours regress upon withdrawal of ectopic c-MYC expression^65^. Even transient inactivation of MYC is sufficient to restore checkpoint mechanisms resulting in tumour regression, remodelling of the tumour microenvironment, and shutdown of angiogenesis^56^. These studies also show that systemic c-MYC inhibition in mice causes a mild and reversible toxicity in proliferative tissues. On the basis of these findings, we analysed whether the *in vivo* oncogenic effects of c-MYC are dependent on BTPF using the *Ela-Myc* model of pancreatic cancer. *KRAS* is the main oncogene involved in human PDAC; however, c-MYC is amplified and overexpressed in 10% and >50% of human PDAC, respectively^66^. The relevance of c-MYC to pancreatic carcinogenesis has been underscored by the finding that, in mice, c-MYC is required for mutant KRas-driven development of preneoplastic pancreatic lesions and invasive tumours^67^. In Ela-Myc mice, dysplasia is observed at 8 weeks of age and multifocal tumours develop starting a few weeks later; acinar tumours occur early while ductal tumours—reminiscent of human PDAC—appear later^42^,^43^. In this highly aggressive model, elimination of a single *Bptf* allele was sufficient to delay tumour initiation (that is, dysplasia) and progression. Importantly, these effects did not result from an incomplete maturation of exocrine cells—as in other systems—since we did not find evidence of altered acinar differentiation in *Bptf*^*P−/−*^ mice, as determined by histological analysis and digestive enzyme mRNA expression. These findings support the notion that disruption of the BPTF-c-MYC interaction may represent a valuable strategy for the therapy of c-MYC-driven tumours. In this regard, it is noteworthy that incomplete inactivation of *Bptf* in acinar cells contributed to tumour development in a fraction of mice. The mechanisms through which *Bptf* inactivation suppresses MYC-driven tumour development *in vivo* need to be studied further.

Despite the *in vivo* genetic evidences, a therapeutic approach to target c-MYC has remained elusive. Unlike other transcription factors, c-MYC lacks a ligand-binding domain, what constitutes a formidable obstacle towards direct inhibition. Recently, the disruption of chromatin-dependent processes that suppress c-MYC activity—such as the inhibition of the BET bromodomain protein Brd4 by JQ1—has shown promising results in experimental models of multiple myeloma, BL, acute myeloid leukaemia, acute lymphoblastic leukaemia and neuroblastoma^68^,^69^. However, it is predicted that the effects of these inhibitors would be restricted to tumours where c-MYC transcription is dependent on BRD4. We have shown that BPTF is required for cell proliferation of BL, PDAC and bladder cancer cells *in vitro*. The broad pattern of expression of BPTF in tissues^70^ and the correlation between BPTF expression and c-MYC signatures in human tumours suggest that a wide range of tumour types might be amenable to the therapeutic disruption of the c-MYC-BPTF axis. We therefore propose that small molecules capable of disrupting this interaction may represent a valuable approach to treat c-MYC-addicted tumours.

To conclude, we have identified BPTF as an important cofactor required for the full deployment of the biological activity of c-MYC *in vitro* and *in vivo*, including its oncogenic effects.

## Methods

### Generation of polyclonal anti-BPTF anti-sera

The RLHRMTSIEREEKEKVKKKEKKQEEETC peptide (residues 913–942) was chemically synthesized, coupled with keyhole limpet haemocyanin (KLH), and used as immunogen for the generation of polyclonal antibodies against BPTF. Two rabbits were inoculated subcutaneously with 500 μg of peptide–KLH conjugate emulsified in Freund's complete adjuvant. Five rounds of 250 μg peptide–KLH boosters were administered together with Freund's incomplete adjuvant to each animal in the interval of 3 weeks. Antibodies against BPTF were purified from serum by affinity chromatography on a HiTrap NHS-activated High Performance column (Sigma-Aldrich, GE17-0716-01, St Louis, MO, USA) and tested by enzyme-linked immunosorbent assay, in HEK293T-BPTF-Flag-transfected cells and in BPTF-silenced VM-CUB-3 cells. Uncropped western blots are provided in Supplementary Fig. 5.

### Cell culture

Primary neonatal HFFs (a kind gift of M. S. Soengas, CNIO, Madrid, Spain), 293T (transformed human embryonic kidney cells, from ATCC, Rockville, MD, USA) and human adherent cancer cells—MIA PaCa-2 (from ATCC), PK9 (pancreas, from C. Iacobuzio-Donahue, Memorial Sloan-Kettering Cancer Center, New York, NY, USA) and VM-CUB-3 (bladder, L. J. Old, Memorial Sloan-Kettering Cancer Center)—were cultured in DMEM (Sigma-Aldrich) supplemented with 10% FBS (HyClone, Logan, UT, USA), sodium pyruvate (Life Technologies, Madrid, Spain) and penicillin/streptomycin (Life Technologies). Mouse *Bptf*^+/+^ and *Bptf*^lox/lox^ MEFs were cultured in DMEM supplemented with 10% FBS, sodium pyruvate, non-essential amino acids (Life Technologies), β-mercaptoethanol (Sigma-Aldrich) and penicillin/streptomycin. NAMALWA and RAJI cells (from ATCC) were cultured in suspension in RPMI medium (Sigma-Aldrich) supplemented with 10% FBS and penicillin/streptomycin.

MEFs were generated by mechanical disruption and trypsin digestion of E13.5 embryos from which the fetal liver and the head had been removed. Recombination efficiency of exon 2 upon Cre recombinase expression was evaluated by PCR on genomic DNA as reported elsewhere^1^ (Supplementary Fig. 1c). The following primers were used: 5′-CTCAGGAATTAAGAGGTAATTGACTATC-3′, 5′-TGATTTAGTTCTGATTGTTAGGTCTAC-3′ and 5′-AGACCAGCCTGTTCTACATGGCCAGCC-3′.

### Animal experiments

The following mouse strains were used: *Bptf*^*lox/lox*^ (ref. 16), *Ptf1a-Cre* (ref. 71), *Ptf1a-CreERT2*^*+/KI*^ (ref. 72) and *Ela1-Myc* (ref. 42). C57BL/6 *Bptf*^*lox/lox*^ mice were obtained from Jackson Laboratories (stock number 009367). Other strains were available at CNIO.

To inactivate *Bptf* to analyse its role in c-MYC-driven pancreatic tumorigenesis, we administered 25 mg of tamoxifen (Sigma-Aldrich, T-5648) by gavage over the course of 1 week to 5–7-week-old *Bptf*^*lox/lox*^*;Ptf1a-CreERT2*^*+/KI*^*;Ela1-Myc* mice and their corresponding controls. Mice were screened for pancreatic tumours once a week using a small animal ultrasound system.

Mice were housed under specific pathogen-free conditions according to institutional guidelines. Mice were observed on a daily basis and killed when they showed signs of morbidity or tumour burden was >10% body weight in accordance with the Guidelines for Human Endpoints for Animals Used in Biomedical Research. All experiments were performed in accordance with the guidelines for Ethical Conduct in the Care and Use of Animals as stated in The International Guiding Principles for Biomedical Research involving Animals, developed by the Council for International Organizations of Medical Sciences (CIOMS), and were approved by the Instituto de Salud Carlos III Ethical Committee.

### Plasmids and lentiviral infections

Mission shRNAs (Sigma-Aldrich) were used for RNA interference. Two BPTF-targeting shRNAs (shBPTF-1, clone TRCN0000016819; shBPTF-2, clone TRCN0000016820) were used and compared with a control non-targeting shRNA. MYC-ER was expressed from the cDNA cloned in FG12 plasmid. For lentiviral transduction of Cre recombinase, we used the lentiviral vector pLVXpuro-iCRE-ORF, a gift from C. Bar and M.A. Blasco (CNIO).

Infectious lentiviruses were produced in 293T cells by calcium phosphate-mediated transfection of the lentiviral construct together with the packaging plasmids psPAX2 and pCMV-VSV-G. Post transfection (48 h), the medium was collected twice for an additional 48 h. Viral supernatants were filtered and either frozen down in aliquots or applied on target cells in the presence of 5 μg ml^−1^ polybrene. Cells were used after 48 h puromycin selection (2 μg ml^−1^). Human fibroblasts were infected first with lentivirus coding for MYC-ER, expanded and then infected with either control or BPTF-targeting shRNAs. MEFs were infected concomitantly with lentivirus encoding for MYC-ER and Cre recombinase.

### Viability assays of BL cell lines

BL cells (3 × 10^5^ cells per well) were seeded on plastic plates coated with retronectin (Fisher Scientific, Pittsburgh, PA, USA) and preloaded with viral supernatants. After three additional rounds of infection with viral supernatants supplemented with polybrene (8 μg ml^−1^), cells were allowed to recover for 24 h, then selected for 48 h in puromycin-containing medium (2 μg ml^−1^). After selection, cells (sh#1, sh#2 and shNT) were plated (5 × 10^3^ per well in 96-well plates) in replicates. Viable cell count was assessed at the indicated time points by adding WST1 cell proliferation reagent (Roche, Basel, Switzerland) to each well and determining OD_450 nm_ after 2 h, according to the manufacturer's instructions.

### Co-immunoprecipitation analyses

293T cells transiently transfected with the corresponding plasmids were washed twice with ice-cold PBS and lysed for 30 min on ice with NP-40 lysis buffer (50 mM Tris:HCl pH 8.0, 150 mM NaCl and 1.0% NP-40) supplemented with a protease inhibitor cocktail. Lysates were then centrifuged at 16,000*g* for 20 min at 4 °C. Total protein (1 mg) was incubated with primary antibody (2 μg) overnight. Protein A/G agarose beads (Laboratorios Conda, Madrid, Spain) preblocked with bovine serum albumin (BSA) were then added to the lysates. Following 4-h incubation at 4 °C, beads were washed three times with NP-40 lysis buffer and immunoprecipitated proteins were eluted with SDS sample buffer by boiling at 90 °C. SDS–polyacrylamide gel electrophoresis was then performed on 6 and 10% (w/v) gels and proteins were then transferred onto nitrocellulose membranes.

### Western blotting

Cells were lysed in RIPA buffer supplemented with protease and phosphatase inhibitors. Following sonication, clearing by centrifugation, and protein determination, equal amounts of protein per sample were subjected to electrophoresis in 8 or 10% polyacrylamide SDS gels, or in NuPAGE 3–8% Tris-acetate precast polyacrylamide gels (Life Technologies). Samples were run under reducing conditions and then transferred to nitrocellulose membranes, which were blocked with TBST, 5% skim milk. Membranes were subsequently incubated with the following primary antibodies: BPTF (1:500; ab72036, Abcam, Cambridge, UK), c-MYC (1:500; 06–340, Millipore, Billerica, MA, USA) and Vinculin (1:2,000; V9131-2ML, Sigma-Aldrich). This was followed by incubation with horseradish peroxidase-conjugated secondary antibodies (1:10,000; Dako, Glostrup, Denmark). Reactions were detected using the ECL system.

### Immunofluorescence staining and isPLA

Cells grown on coverslips were fixed with 4% paraformaldehyde for 10 min, washed and permeabilized with 0.1% Triton X-100 in PBS for 10 min. Samples were washed in PBS and blocked with 3% BSA in PBS for 1 h at room temperature. Primary antibody incubation was performed in blocking solution for 2 h at room temperature. Mouse anti-MYC (C-33, sc-42, Santa Cruz Biotechnology, Dallas, TX, USA) was used at a 1:50 dilution and home-made affinity-purified rabbit anti-BPTF antibodies were used at 10 μg ml^−1^. After three washes with PBS, cells were incubated with an appropriate secondary antibody diluted in blocking solution. Nuclei were counterstained with DAPI and coverslips were mounted on ProLong (Life Technologies). Images were taken with a confocal microscope, using a × 40 immersion oil lens. For the isPLA, the DuolinkII fluorescence system was used (Olink Bioscience, Uppsala, Sweden).

### Quantitative real-time PCR

Total RNA was isolated from cultured cells using the GenElute Mammalian Total RNA Miniprep Kit (Sigma-Aldrich) according to manufacturer's instructions. Samples were treated with DNase I before reverse transcription (Life Technologies). cDNA was generated from 1 μg of RNA using random hexamers and reverse transcriptase. Real-time PCR amplification and analysis was conducted using the 7900HT Real-Time PCR System (Applied Biosystems, Life Technologies). RNA levels were normalized to GAPDH expression using the ΔΔCt method. For RT–qPCR analysis, primer pairs were designed to achieve product lengths of 200–250 bp. Primer sequences are provided in Supplementary Data 3.

### FACS analysis of proliferation and apoptosis

For cell cycle analysis, cells were pulse labelled with 10 μM of BrdU (Sigma-Aldrich) for 1 h, collected by trypsinization and then fixed in 100% ethanol. Upon DNA denaturation using 2 N HCl, cells were stained with mouse anti-BrdU primary antibody (sc-51514, Santa Cruz Biotechnology; 1 μg per 10^6^ cells) and anti-mouse Alexa Fluor 488-conjugated secondary antibody (A21202, Life Technologies; 1 μg per 10^6^ cells). DNA was stained by resuspending cells in 0.1 mg ml^−1^ of propidium iodide and incubating for 30 min at room temperature until fluorescence-activated cell sorting (FACS) analysis.

To measure apoptosis, MEFs were seeded at high density and then transferred to 0.5% FBS-containing DMEM in the presence of either vehicle (EtOH) or 2 mM 4-OHT. At the indicated time points, cells and supernatants were collected, washed and resuspended in Annexin V-binding buffer containing 5 μl per sample of Annexin V-APC (550474, BD Biosciences, Franklin Lakes, NJ, USA). DAPI was added before analysis.

All samples were analysed using a FACS Canto II (BD Biosciences) flow cytometer. At least 10,000 events were acquired. Analyses were performed using FlowJo flow cytometry anaysis software.

### Chromatin immunoprecipitation

Cells were fixed with 1% formaldehyde for 15 min at room temperature. Fixation was stopped by adding glycine (to 0.125 M) with an additional incubation of 5 min. Cells were collected by scraping, pelleted and then lysed for 10 min in 1 ml of buffer LB1 (50 mM HEPES (pH=7.5), 140 mM NaCl, 1 mM EDTA, 0.5% NP-40, 0.25% Triton X-100 and 10% glycerol) supplemented with protease inhibitors (Qiagen, Valencia, CA, USA). After centrifugation at 3,000*g*, pelleted nuclei were resuspended in 1 ml of buffer LB2 (10 mM Tris (pH=8.0), 200 mM NaCl, 0.5 mM EGTA and 1 mM EDTA), and incubated at room temperature for 10 min. Pelleted nuclei were resuspended in 1 ml of ChIP SDS buffer (100 mM NaCl, 50 mM Tris (pH 8), 5 mM EDTA pH 8, 0.2% NaN_3_ and 0.5% SDS) and sonicated for 20 min in a Covaris sonicator, yelding DNA fragments of 300–500 bp. Beads were blocked overnight in PBS with 0.5% BSA and then added to the samples. After a 3-h incubation at 4 °C, beads were washed with Triton dilution buffer (100 mM Tris (pH=8.6), 100 mM NaCl, 5 mM EDTA (pH=8), 0.2% NaN_3_ and 5% Triton X-100), mixed micelle wash buffer (150 mM NaCl, 20 mM Tris (pH 8), 5 mM EDTA (pH 8), 5% sucrose, 0.2% NaN_3_, 1% Triton X-100 and 0.2% SDS), Buffer 500 (0.1% deoxycholic acid, 1 mM EDTA (pH=8), 50 mM HEPES (pH=7.5), 1% Triton X-100, 500 mM NaCl and 0.2% NaN_3_), LiCl buffer (0.5% deoxycholic acid, 1 mM EDTA (pH 8), 250 mM LiCl, 0.5% NP-40, 10 mM Tris (pH=8) and 0.2% NaN_3_) and Tris-EDTA (TE). DNA was eluted in elution buffer and crosslinks were reversed by incubation overnight at 65 °C. RNA and protein were digested using RNase A and Proteinase K and DNA was purified by phenol–chloroform extraction and isopropanol precipitation. Target DNA abundance in ChIP eluates was assayed by qPCR with primer pairs designed to achieve products of 50–200 bp. Primer sequences are provided in Supplementary Data 3. The following antibodies were used: anti-MYC N262 (sc-764, Santa Cruz Biotechnology), anti-H3K4me3 (ab8580, Abcam), anti-panAc Histone H3 (06–599, Merk Millipore) and anti-Histone H3 (ab1791, Abcam).

### DNase I hypersensitivity assay

Chromatin samples were subjected to DNAse I digestion. Chromatin (2 μg) was treated with 0.5, 1 and 2 units of RQ1 RNase-Free DNAse I (Promega, Fitchburg, WI, USA) for 3 min at 37 °C in 1 × DNAse incubation buffer. Reactions were terminated by adding 2 mM EGTA and the crosslinking was reversed by incubating samples at 65 °C. After 6 h, proteinase K (40 mg ml^−1^) was added to each reaction and incubated overnight at 37 °C. After phenol–chloroform extraction, DNA was quantified and used as template for qPCR reactions with the same primer pairs used for ChIP–qPCR.

### ChIP-Seq libraries and massive parallel sequencing

ChIP was performed as described above. DNA (20 ng) was quantified by fluorimetry, resolved by electrophoresis and fractions of 50–250 bp were extracted. Input samples correspond to balanced blends of inputs from selected samples. Fractions were processed through subsequent enzymatic treatments of end repair, dA tailing and ligation to adaptors following Illumina's ‘TruSeq DNA Sample Preparation Guide' (part #15005180 Rev. C). Adaptor-ligated libraries were amplified by limited-cycle PCR with Illumina PE primers (12 cycles). The resulting purified DNA library was applied to an Illumina flow cell for cluster generation (TruSeq cluster generation kit v5) and sequenced on the Genome Analyzer IIx with SBS TruSeq v5 reagents following the manufacturer's protocols.

### ChIP-Seq data processing

Image analysis and per-cycle base calling was performed with Illumina Real Time Analysis software (RTA1.13). Conversion to FASTQ read format with the ELAND algorithm (v2e) was performed with CASAVA-1.8 (Illumina). Quality check was done via fastqc (v0.9.4, Babraham Bioinformatics). ChIP-Seq reads were aligned to the human reference genome (GRCh37/hg19, February 2009) with Burrows-Wheeler Aligner (v0.5.9-r16) allowing 0–1 mismatches. Unique aligned reads were converted to BED format (Supplementary Data 4). All ChIP and input samples were normalized randomly to the same number of reads (10,512.988). Furthermore, reads were directionally extended to 300 bp and, for each base pair in the genome, the number of overlapping sequence reads was determined and averaged over a 10-bp window to create a wig file to visualize the data in the University of California Santa Cruz genome browser. The number of significant peaks of MYC-binding sites was 1,762 for sh#1+OHT and 1,397 for shNt+OHT, using MACS (version 2.0.9 20111102, tag:alpha) and parameters: −g 2.7e9; −m 10,30; −q 0.05.

### Motif-enrichment analysis

Motifs for the list of peaks in shNt+OHT were identified with the MEME suite and then TOMTOM was used to compare the identified motifs with known transcription factor-binding motifs. Sequence logos were generated using WebLogo 2.8.2.

### Peak annotation and density plot analysis

Genomic annotation was carried out with Hypergeometric Optimization of Motif EnRichment (software v4.2). The tool annotatePeaks.pl was used with parameters by default and defined in the help. A gtf file from University of California Santa Cruz based on GRCh37/hg19 was used for annotations; the latter included whether a segment is in the TSS, transcription termination site, exon, 5′-untranslated region (UTR) exon, 3′-UTR exon, intron or is intergenic. Since some annotations overlap, the following priority was assigned: TSS (from −1 kb to +100 bp), transcription termination site (from −100 bp to +1 kb), protein coding exon, 5′-UTR exon, 3′-UTR exon, intron and intergenic. More detailed information is available in http://homer.salk.edu/homer/ngs/annotation.html. The SeqMINER (v1.3.3e) platform^73^ was used to generate the density read plots shown in Supplementary Fig. 3a.

### Gene Set Enrichment Analysis

MYC-bound genes were rank-ordered according to the fold change in FPKM (fragments per kilobase of exon per million fragments mapped) values (4-OHT versus vehicle) in HFF MYC-ER control cells and then submitted to analysis using GSEA software (www.broadinstitute.org/gsea). The list of pre-ranked genes was analysed with the gene set matrix composed file c2.all.v4.0.symbols.gmt and c5.all.v4.0.symbols.gmt. Significant gene sets enriched by 4-OHT treatment of control cells were identified using a false discovery rate q-value<0.25 and a nominal *P* value<0.05, as defined by http://www.broadinstitute.org/gsea/doc/GSEAUserGuideFrame.html?Interpreting_GSEA.

### RNA-seq

Total RNA (1 μg) was spiked with ERCC ExFold RNA spike-In mixes (Life Technologies). RNA quality was assessed on an Agilent 2100 Bioanalyzer and samples with a RNA integrity number >8.5 were used. PolyA+ fractions were purified, randomly fragmented, converted to double-stranded cDNA and processed through subsequent enzymatic treatments of end repair, dA tailing and ligation to adaptors following Illumina ‘TruSeq Stranded mRNA Sample Preparation Part # 15031047 Rev. D' (this kit incorporates dUTP during second-strand cDNA synthesis, which implies that only the cDNA strand generated during first-strand synthesis is eventually sequenced). Adaptor-ligated libraries were generated by PCR with Illumina PE primers (eight cycles). The resulting purified cDNA libraries were applied to an Illumina flow cell for cluster generation (TruSeq cluster generation kit v5) and sequenced on the Genome Analyzer IIx with SBS TruSeq v5 reagents by following the manufacturer's protocols.

### RNA-Seq data processing

Image analysis and per-cycle base-calling was performed with Illumina Real Time Analysis software (RTA1.13). Conversion to FASTQ read format with the ELAND algorithm (v2e) was performed with CASAVA-1.8 (Illumina). These files contain only reads that passed ‘chastity' filtering (flagged with a ‘N', for ‘not filtered' in the sequence identifier line). ‘Chastity' parameter measures signal contamination in raw data and allows to flag unreliable reads. Quality check was done via fastqc (v0.9.4, Babraham Bioinformatics). Raw reads were aligned to the build version GRCh37/hg19 of the human genome where the sequence of the ERCC synthetic spike-in RNAs (http://tools.invitrogen.com/downloads/ERCC92.fa) had been added. Tophat5 (version 2.0.4) was used for alignment with the following parameters: --bowtie1, --max-multihits 5, --genome-read-mismatches 1 --segment-mismatches 1 --segment-length 19 --splice-mismatches 0 --library-type fr-firststrand. Gene expression levels and synthetic spike-in RNA (FPKM) were quantified with cufflinks (version 2.0.2), with the following parameters: -N, --library-type fr-firststrand, -u. Further, we used a loess regression to renormalize the FPKM values by using only the spike-in values to fit the loess following the strategy described^74^. The *affy* package in R provides a function, loess.normalize, performing loess regression on a matrix of values and allowing to specify which subset of data to use when fitting the loess (see the *affy* package for further details). The result was a matrix of FPKM values normalized to the control ERCC spike-ins.

### RNA-Seq GSEA analysis

Genes were rank-ordered according to the fold change in FPKM values (4-OHT versus vehicle) in HFF MYC-ER control cells and then submitted to analysis using GSEA software (www.broadinstitute.org/gsea). The list of pre-ranked genes was analysed with the gene set matrix composed file c2.all.v4.0.symbols.gmt and c5.all.v4.0.symbols.gmt. Significant gene sets enriched by OHT treatment of control cells were identified using a false discovery rate q-value<0.25 and a nominal *P* value<0.05. All analyses were performed using GSEA v2.1 software with pre-ranked list and 1000 data permutations.

### Analysis of human genomic tumour data

Gene expression data from 20 studies profiling human tumours were downloaded from either Oncomine or GEO. The complete list of data sets, together with their GEO accession numbers, is provided in Supplementary Data 9. Expression data for each study were converted into the GenePattern GCT format. To obtain one expression value per gene and sample, GCT files were subsequently collapsed using the CollapseDataset module in GenePattern. We next rank-ordered the samples within each data set according to the mRNA levels of BPTF, c-MYC, N-MYC or L-MYC and performed a ssGSEA to calculate activation scores for four MYC-dependent gene signatures in each sample. ssGSEA-enrichment score represents the degree to which the genes in a particular gene set are coordinately up- or downregulated within a sample. The following gene signatures were downloaded from Molecular Signature Database: BILD_MYC_ONCOGENIC_SIGNATURE (M2069), ALFANO_MYC_TARGETS (M2477) and SCHUHMACHER_MYC_TARGETS_UP. The Seitz signature was built from the data published in ref. 75.

### Statistical analysis

All quantitative data are presented as mean±s.e.m. from ≥3 experiments or samples per data point (*n* is mentioned in each figure legend). Unpaired Student's *t*-test (two-tailed) was used to compare two groups of independent samples. Paired Student's *t*-test (two-tailed) was used to compare matched pairs samples. To compare the data distribution of two separate populations without assuming normal distribution, we performed a Wilcoxon signed-ranked test. For *in vitro* experiments, sample size required was not determined *a priori*. The experiments were not randomized.

## Additional information

**Accession codes:** ChIP-Seq and RNA-Seq Data sets have been deposited at GEO with the following accession numbers GSE65544 (ChIP-Seq), GSE65545 (RNA-Seq) and GSE65546 (the combined data set).

**How to cite this article:** Richart, L. *et al.* Bptf is required for c-MYC transcriptional activity and *in vivo* tumorigenesis. *Nat. Commun.* 7:10153 doi: 10.1038/ncomms10153 (2016).

## Supplementary Material

Supplementary Information Supplementary Figures 1-5

Supplementary Data 1 Expression data for all genes sequenced in HFF MYC-ER.

Supplementary Data 2 GSEA analysis of control HFF MYC-ER cells.

Supplementary Data 3 RT-qPCR and ChIP-qPCR primers.

Supplementary Data 4 Results of the ChIP-seq experiment with c-MYC antibodies.

Supplementary Data 5 GSEA analysis of c-MYC-bound promoters in control HFF MYC-ER cells.

Supplementary Data 6 MYC binding intensity.

Supplementary Data 7 Validation ChIP-seq experiment.

Supplementary Data 8 DNAse hypersensitivity data.

Supplementary Data 9 Summary human studies.

Supplementary Data 10 Gene sets.
