# Supplementary material for: BPTF is required for c-MYC transcriptional activity and in vivo tumorigenesis
Source: Nat Commun. 2016 Jan 5;7:10153. doi: 10.1038/ncomms10153 (PMC4728380; doi:10.1038/ncomms10153)
Supplement: Supplementary Information — Supplementary Figures 1-5 [file ncomms10153-s1.pdf]

Supplementary Figure 1

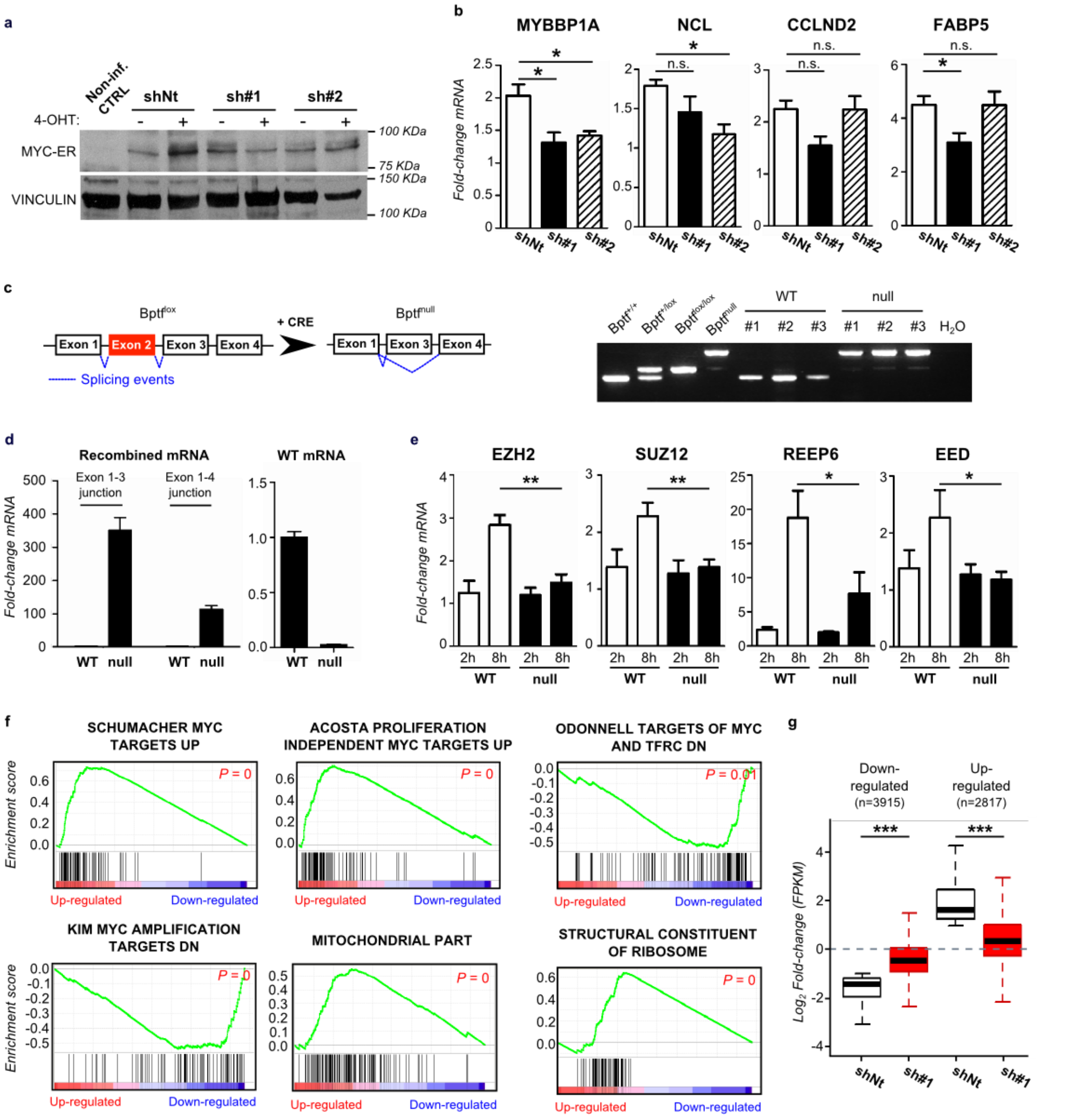

Supplementary Figure 1. *BPTF is required for c-MYC transcriptional activity*. (a) Western blot analysis of control and BPTF-silenced HFF transduced with MYC-ER fusion protein. (b) Expression of a panel of c-MYC target genes. mRNA levels were analysed by RT-qPCR and normalized to GAPDH and the vehicle-treated condition. Error bars represent the mean±SEM (Standard Error of Mean) of at least 5 independent experiments. *P* values were determined by unpaired *t*-tests. (c) Diagram showing the *Bptf* floxed allele and assessment of Cre-mediated recombination at the DNA level. (d) Upon recombination, two mutant mRNA species can be specifically detected in *Bptf*-null MEFs by RT-qPCR. (e) Expression of a set of c-MYC target genes in WT and *Bptf*-null MEFs (n ≥ 4) expressing MYC-ER. Cells arrested with 0.5% FBS for 24h were treated for the indicated time with 10% FBS with or without 4-OHT 2 μM. Data are represented as the mean±SEM. *P* values were determined using unpaired *t*-tests. (f) Snapshots of MYC-dependent gene sets displaying either a positive or a negative enrichment in 4-OHT-treated control cells. (g) Graph plotting fold-change in FPKM values (vehicle vs. 4-OHT) of up-regulated [Log_2 F.c. ≥ +1] and down-regulated [Log_2 F.c. ≤ -1] genes in control and BPTF-silenced cells. *P* value was determined using a Wilcoxon test. *, *P* value < 0.05. **, *P* value < 0.01. ***, *P* value < 0.001.

Supplementary Figure 2

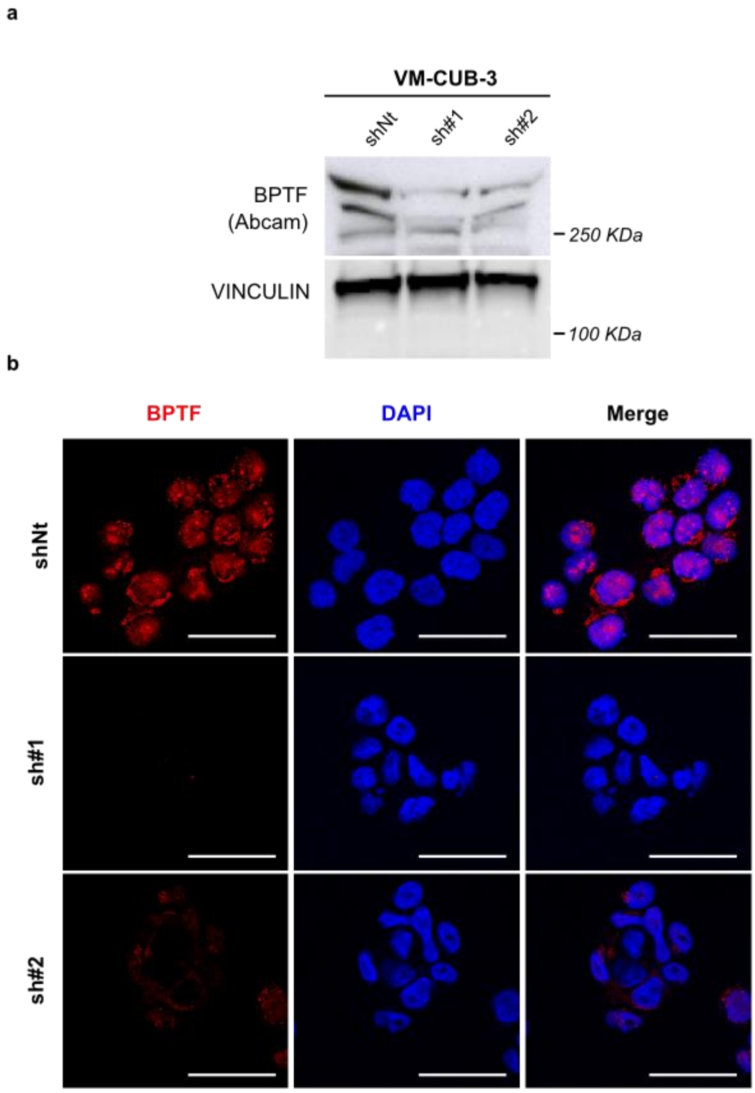

Supplementary Figure 2. *Specificity of anti-BPTF antibodies*. (a) Western blot showing effective BPTF down-regulation in VM-CUB-3 cells with a commercial anti-BPTF antibody. (b) Immunofluorescence and DAPI staining of control (shNt) and BPTF-silenced (sh#1 and sh#2) VM-CUB-3 cells showing specific staining with the home-made affinity-purified antibodies recognizing residues 913-942 of BPTF.

Supplementary Figure 3

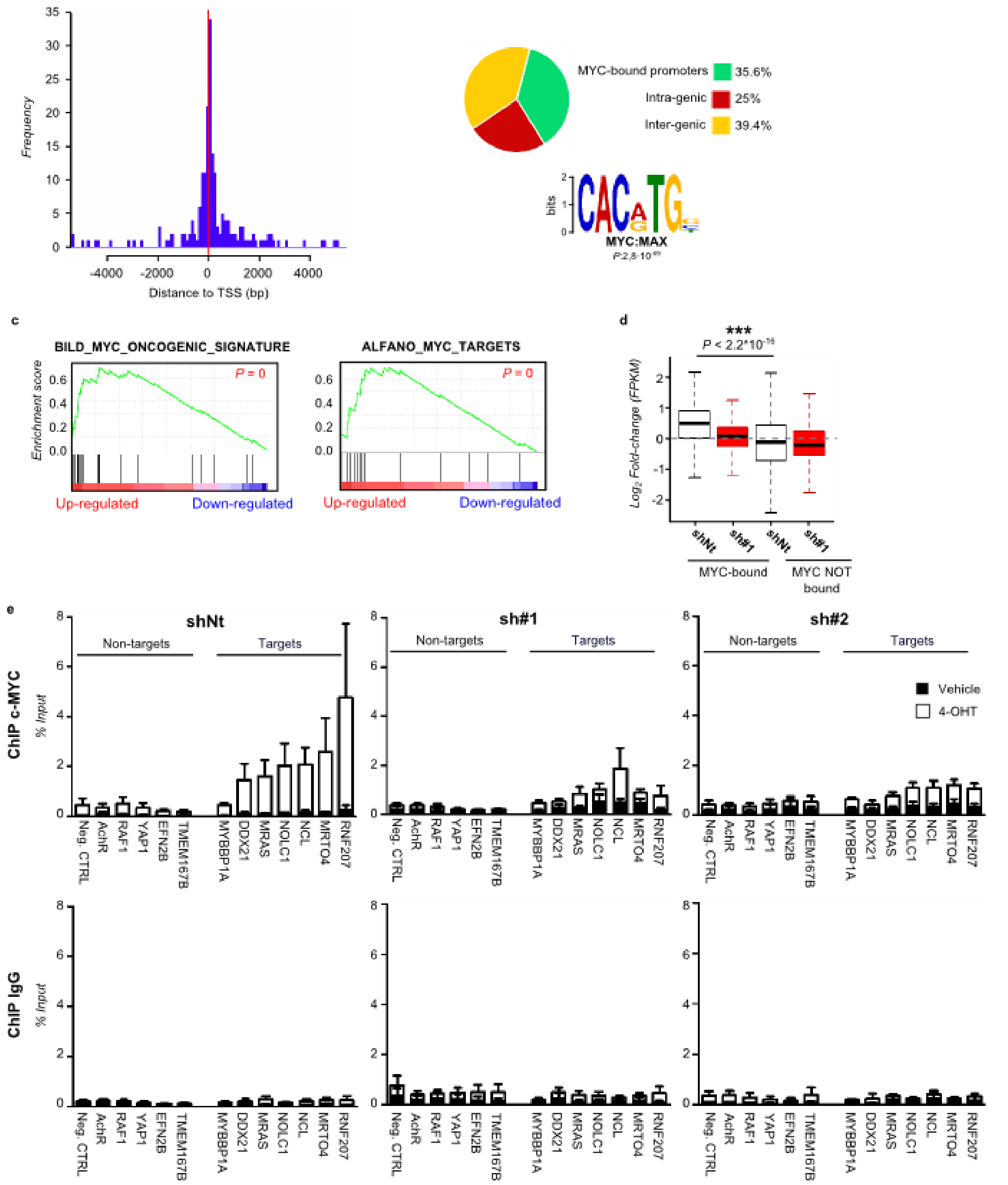

Supplementary Figure 3. *Analysis of MYC-ER recruitment to chromatin.* (a) Density profile of c-MYC binding sites relative to TSS. All binding sites within ± 6kb were included in the analysis. TSS distance is measured as the relative base pair distance to peaks' summit. (b) Distribution of c-MYC binding sites relative to the gene bodies of Ref Seq annotated transcripts. MEME motif prediction of DNA sequences enriched in c-MYC ChIPseq in 4-OHT-treated shNt cells. (c) Snapshots of c-MYC-dependent gene sets enriched among c-MYC-bound promoters in control cells. (d) Fold-change in FPKM values (4-OHT vs vehicle) for genes bound by c-MYC (with a ChIP-Seq peak within ±3kb TSS) and genes not bound, both in control and BPTF-silenced cells. ***, *P* value < 0.001 (Wilcoxon test). (e) An isotype-matched IgG was used as control in the ChIP experiments conducted in control and BPTF-silenced HFF MYC-ER cells (referred to Fig. 2c). ChIP values are expressed as average±SEM of % input chromatin.

Supplementary Figure 4

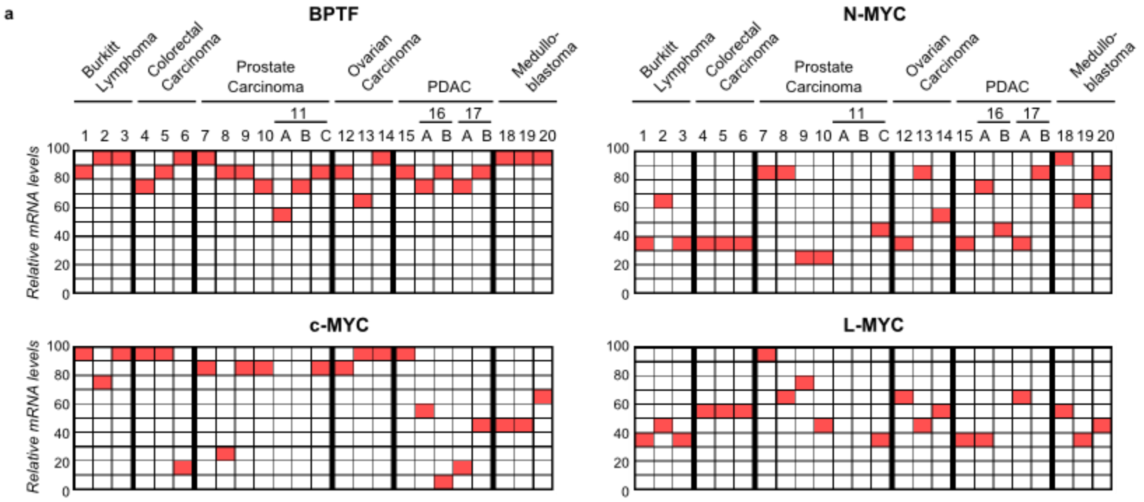

Supplementary Figure 4. *BPTF and MYC genes expression in human tumors*. (a) Position of *BPTF*, *c-MYC*, *MYCN* and *MYCL1* mRNAs within the lists of transcribed genes rank-ordered by their expression values in a collection of human tumors. The details of each study are provided in Supplementary Data 9.

Supplementary Figure 5

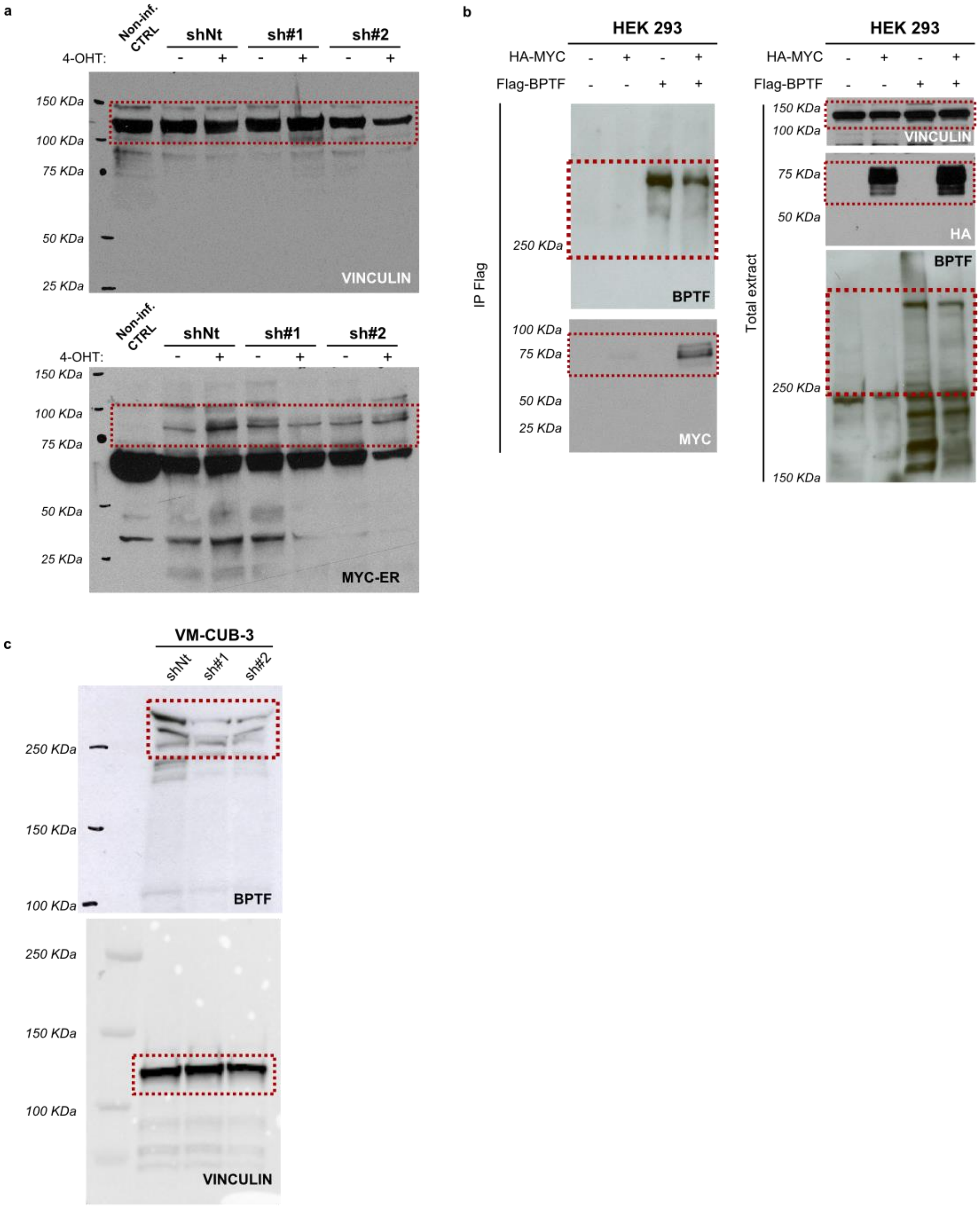

Supplementary Figure 5. Original figures for Western Blots in Supplementary Figure 1a (a), Figure 1f (b), and Supplementary Figure 2a (c).
